# Molecular epidemiology to aid virtual elimination of HIV transmission in Australia

**DOI:** 10.1016/j.virusres.2024.199310

**Published:** 2024-01-11

**Authors:** Billal M. Obeng, Anthony D. Kelleher, Francesca Di Giallonardo

**Affiliations:** The Kirby Institute, University of New South Wales, Sydney, Australia

**Keywords:** HIV, Prevention, Molecular epidemiology, Phylogenetics, Transmission network, Surveillance

## Abstract

The Global UNAIDS 95/95/95 targets aim to increase the percentage of persons who know their HIV status, receive antiretroviral therapy, and have achieved viral suppression. Achieving these targets requires efforts to improve the public health response to increase access to care for those living with HIV, identify those yet undiagnosed with HIV early, and increase access to prevention for those most at risk of HIV acquisition. HIV infections in Australia are among the lowest globally having recorded significant declines in new diagnoses in the last decade. However, the HIV epidemic has changed with an increasing proportion of newly diagnosed infections among those born outside Australia observed in the last five years. Thus, the current prevention efforts are not enough to achieve the UNAIDS targets and virtual elimination across all population groups. We believe both are possible by including molecular epidemiology in the public health response. Molecular epidemiology methods have been crucial in the field of HIV prevention, particularly in demonstrating the efficacy of treatment as prevention. Cluster detection using molecular epidemiology can provide opportunities for the real-time detection of new outbreaks before they grow, and cluster detection programs are now part of the public health response in the USA and Canada.

Here, we review what molecular epidemiology has taught us about HIV evolution and spread. We summarize how we can use this knowledge to improve public health measures by presenting case studies from the USA and Canada. We discuss the successes and challenges of current public health programs in Australia, and how we could use cluster detection as an add-on to identify gaps in current prevention measures easier and respond quicker to growing clusters. Lastly, we raise important ethical and legal challenges that need to be addressed when HIV genotypic data is used in combination with personal data.

## Highlights

• Australia can achieve virtual elimination of HIV through molecular epidemiology.
• Major drivers of HIV transmission are early infection and the undiagnosed.
• Prevention programs are tailored for different risk population groups.
• Ethical implications critical for public health use of molecular epidemiology.

## 1 Background

In 2022, an estimated 39 million people worldwide were living with HIV (UNAIDS 2023), including 27,390 in Australia (Kirby 2022). Here, the epidemic was first recognised in the early 1980s, and soon after, the number of new infections reported rose rapidly (Grulich and Kaldor, 2008). To address this crisis, various public health interventions have been implemented and then adapted to changes in circumstances over time resulting in decreased transmission rates (Kirby, 2023). Initially, these interventions focused on promoting safe sex practices and establishing needle exchange programs. Eventually, with the development and scale-up of effective antiretroviral therapies (ART), “Treatment as Prevention” (TasP) and pre-exposure prophylaxis (PrEP) programs were introduced and incorporated into national HIV prevention initiatives to help decrease the rate of new HIV infections (Bavinton et al., 2018; Grant et al., 2010; Bavinton and Grulich, 2021). Notably, the number of new HIV diagnoses has almost halved in the last ten years, from 1,068 new infections reported in 2012 to 555 in 2022 (Kirby, 2022, 2023).

Nevertheless, gaps in uptake of prevention methodologies persist, forestalling further significant reductions in HIV transmission rate in certain populations and geographic areas. For example, among persons born in North Africa and Middle East, the number of HIV notifications increased from 1.3 in 2019 to 2.3 in 2021 per every 100,000 persons (Kirby, 2022). Most persons were reported to have acquired HIV in Australia (59%) compared to those who acquired it overseas (29%) with unknown place of acquisition as 12% (Kirby, 2022). During the same period, Indigenous Australians who inject drugs were disproportionately affected by HIV, with diagnosis rates more than three times higher (11.3%) than non-indigenous Australians in the same at-risk group (2.3%) (Kirby, 2022). To maximize efforts, multifaceted approaches to the study and understanding of HIV transmission dynamics are needed in order to reduce these residual new HIV infections more efficiently. To do this, some developed countries have added molecular epidemiology to their prevention strategies (Oster et al., 2018). Such surveillance can benefit from sequences generated through routine HIV genotypic antiretroviral testing (GART) being linked to an individual's data including demographic, behavioral, clinical, serologic, risk factor and geographic parameters to better inform public health strategies (Rich et al., 2020; Mazrouee et al., 2021; Aldous et al., 2012). This allows better understanding of the location and behavioural determinants of ongoing transmission.

## 2 Advances in HIV prevention in Australia

**Fig. 1:**
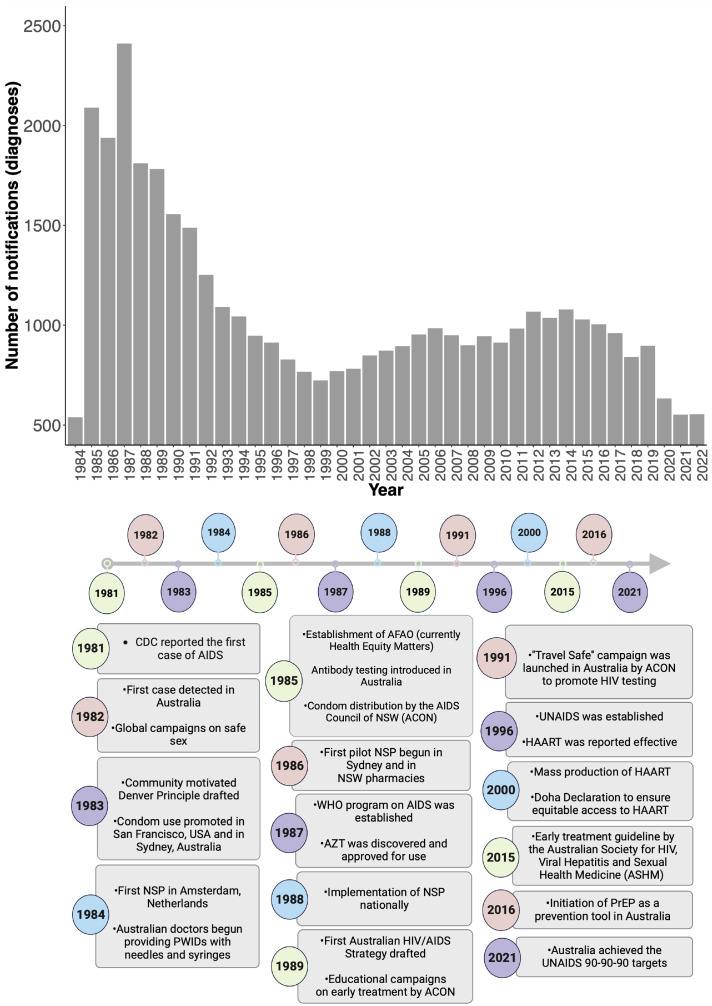
Key historical efforts in HIV/AIDS prevention. Top panel: Number of new HIV notifications (diagnoses) on a national basis for Australia. Bottom panel: Several prevention efforts have been pioneered by federal governments and the community since the first case of HIV in 1981. Australia has since introduced interventions from structural provisions like the establishment of AFAO and State HIV strategies to biological interventions such as equitable access to ARTs for treatment and PrEP. Abbreviations: ACON = AIDS council of NSW, NSW = New South Wales, NSP = needle and syringe program, HARRT = highly active antiretroviral therapy, PrEP = Pre-exposure prophylaxis.

In Australia, HIV notifications (diagnoses) peaked in 1987 with approximately 2,500 new diagnoses reported that year (Fig. 1) (Kaldor, 1993). This peak followed the introduction of systematic laboratory testing for HIV in 1985 (Kaldor, 1993; Whyte et al., 1987). Community-based AIDS councils were established across different States and Territories following the diagnosis of the first case in 1982. These councils led the promotion of peer education on HIV testing and prevention. Through funding from the Australian government, the Australian Federation of AIDS Organisations (AFAO, now known as Health Equity Matters) was established in 1985, which became the national body representing HIV community organisations and lead the think-tank that helped develop the first National HIV/AIDS strategy in 1989 (AFAO, 2017). Co-ordinated community involvement in the response has been a pillar of HIV prevention in Australia, through the provision of active input into the programmatic design, implementation, and assessment of HIV prevention policies and strategies developed by the federal and state governments. Through political, social, academic and financial support, success in prevention has been observed in the last decade with steady declines in HIV new diagnoses (Kirby 2022, 2023) (Fig. 1). At the end of 2021, Australia had achieved all aspects of the UNAIDS 90/90/90 targets recording 91% of HIV diagnoses, with 92% treatment of the diagnosed population on treatment and 98% of those having a suppressed viral load.

### 2.1 Safe sex messages

Public health messaging around HIV has evolved, reflecting changes in societal attitudes and advances in HIV prevention. Safe sex messages have been essential in reducing HIV transmission rates since the earliest days of the epidemic in Australia. After the first AIDS diagnosis in Sydney in 1982, efforts were made to raise awareness about the modes of HIV transmission and promote safe sex practices across the population, but especially in key at-risk groups such as men-who-have-sex-with-men (MSM) and sex workers (Penny et al., 1983). Condom use was promoted as an effective barrier method of preventing HIV transmission during sexual intercourse (Roper et al., 1993). In Australia, a third of healthy men and a quarter of men living with HIV, were reported to consistently use condom for anal intercourse within a 6-month period in 2011 (Mao et al., 2011). However, as PrEP usage increased, there has been a corresponding decrease in consistent condom use. From 2013 to 2017, a study conducted in Australia reported significant decline (46% in 2013 to 31% in 2017) in condom use among MSM (Holt et al., 2018). The findings highlight the potential trade-off between PrEP use and condom use among MSM. This adds to the already increasing early continuous use of effective ART in those living with HIV and the acceptance of U = U (Undetectable = Untransmissible), all of which have resulted in refined definitions of safe sex (Holt et al., 2018). However, PrEP use is not uniform across population groups and safe sex messages are influenced by varying levels of stigma and accessibility of information which are also impacted by a variety of factors including cultural diversity. Identifying these gaps and barriers is pivotal for the next steps in effective prevention strategies.

### 2.2 Needle-exchange program

A public needle and syringe program was first started in 1986 in Sydney (Fig. 1) to help reduce transmission among people who inject drugs (PWID). The success of this pilot study led to the program being implemented nationally in 1988 (ANCD, 2014). It was estimated that the needle and syringe program helped prevent about 25,000 HIV infections by 2000 among PWID. Currently, PWID represents only 2.2% of those living with HIV infection and only 1.6% of the new diagnoses in Australia (Kirby, 2022). The needle and syringe program has been critical in reducing HIV notification and limiting its spread, especially from MSM into the heterosexual community (Kirby, 2023). HIV transmission via intravenous drug use is regarded as virtually eliminated (sustained reduction of HIV below endemic levels) in Australia, with less than 30 new cases reported between 2018 and 2022 (Kirby, 2023). Even though needle and syringe programs have been implemented in several countries, their success is often affected by access to sterile needles by the affected populations (WHO, 2007; Stockman and Strathdee, 2010). In Australia, one challenge is effectively reaching marginalised and vulnerable populations, including culturally and linguistically diverse communities, Indigenous Australians and those who are homeless or incarcerated (Poeder, 2013; McDonald, 2007). This requires making needle exchange programs more accessible and tailored to the needs of these populations. For instance, there remains significant resistance to implementation of needle and syringe program in prisons in all jurisdictions in Australia.

### 2.3 Testing

In Australia, one critical step in preventing HIV has been effective and large-scale, freely available testing (AGDH 2014). This is based on voluntary testing among priority populations such as MSM. The public health benefits of early detection rely on maintaining an effective and responsive testing regime through continued review of testing policies including funding and facilitating infrastructure such as community-based drop-in testing sites for rapid testing. In 2021, nine percent of Australians living with HIV were undiagnosed (Kirby, 2022). This manifests in many of these being diagnosed late with a CD4^+^ *T* cell count of <350 cells/μl (Kirby, 2022). Importantly, this group is critical for further reductions in the rates of HIV transmission. In a recent study, uptake of HIV testing among 22,662 Australian-born and 20,834 overseas-born MSM was evaluated (Patel et al., 2021). It found an overall increased uptake of HIV testing (83.9% in 2010 to 95.1% in 2018) and a significant decline in the proportion of undiagnosed HIV among Australian-born MSM (7.1% in 2010 to 2.8% in 2018), but not among overseas-born MSM (15.3% in 2010 to 16.9% in 2018). It is hoped that recent government initiatives facilitating access to self-testing using the Atomo HIV Self-Test will help reduce this gap (AGDH 2021).

### 2.4 Treatment as Prevention (TasP) with facilitated diagnosis and early treatment

The success of antiretrovirals in stopping HIV replication within people living with HIV, resulting in reduced mortality and morbidity, especially when started early in infection (ISSG, 2015), and the improvement in drug efficacy and tolerance have changed the guidelines of treatment initiation such that the start of treatment is now recommended immediately at diagnosis. The simplicity and reduced side effects of modern ART regimens have made ART more acceptable, allowing greater adherence. This enables individuals to quickly suppress their viral load to undetectable levels and thus, become non-infectious. TasP underpins the second pillar of the HIV cascade, and the aim of the current national strategy is to reach suppression in 95% of all those diagnosed and then treated for HIV infection.

TasP with facilitated diagnosis and early treatment involves increasing access to testing while providing early access to therapy to limit the time taken between infection and viral suppression, and thus, prevent transmission. There is robust evidence to suggest that TasP is highly effective in reducing HIV transmission rates (Cohen et al., 2011; Reynolds et al., 2011; Donnell et al., 2010). Thanks to the TasP and prevention of mother-to-child transmission programs, transmission of HIV from mother to child is virtually eliminated in Australia (O'Donovan and Emeto, 2018). The concept of ART reducing viral load to undetectable levels conferring safety from transmission, even in MSM who engage in anal intercourse has been messaged as U = U (Campaign, 2018). This is supported by recent evidence of minimal risk of HIV transmission between serodiscordant couples when the partner living with HIV consistently has viral load of <1000 copies/ml as reported by the World Health Organization (WHO) (Broyles et al., 2023).

### 2.5 Pre-exposure prophylaxis (PrEP)

When linked to appropriate adherence, PrEP is highly effective in reducing new infections most notably among MSM (Bavinton and Grulich, 2021; Hammoud et al., 2019). Since the rapid at scale roll-out of PrEP among MSM in New South Wales in 2016 using well-designed implementation projects (Grulich et al., 2018) and then in other jurisdictions followed by its subsequent approval as a subsidised medicine, Australia has recorded a steady decline in new HIV notifications overall and particularly in MSM (Kirby, 2022). Even though the benefits of PrEP have been associated with very significant reductions in transmission, specifically in Australian-born MSM, there have been challenges associated with the effective integration into clinical settings such as its access by populations outside central urban areas, in recent immigrants and other minority populations, such as those that do not qualify for the publicly-funded health insurance and subsidised supply of approved pharmaceuticals (Smith et al., 2021).

While PrEP has been a game-changer in HIV prevention, it is essential to recognize that it is not a standalone solution. Combining PrEP with other interventions can have a more significant impact on reducing HIV transmission and achieving the goal of virtually eliminating HIV transmission. In inner Sydney, the combination of accessibility to PrEP and early initiation of treatment and rapid viral suppression have resulted in an 88% reduction in new diagnoses of HIV (Kirby, 2023). It is essential to recognize that greater efforts are required to help efficiently target these interventions to outbreaks in an increasingly sporadic epidemic outside the previous epicenter of this epidemic. The advances in genomic epidemiology can help in this regard, enhancing HIV surveillance and guiding targeted public health responses. Molecular epidemiology studies have revealed changing patterns within transmission groups, allowing for targeted interventions and control of distinct sub-epidemics (Di Giallonardo et al., 2019; Castley et al., 2017). These findings inform ongoing discussions about the integration of molecular approaches into the routine characterization of HIV transmission and the shaping of public health responses. Collaboration with affected communities remains critical in the successful implementation of these approaches.

## 3 Methods for HIV molecular epidemiology

Distance based methods and phylogenetics are most commonly used for HIV clustering analysis. Both methods are suitable for RNA viruses, such as HIV-1, that are characterised by smaller genomes and a fast error-prone replication machinery causing high genetic diversity (Lemey et al., 2006). Distance based methods such as that employed in *HIV-TRACE* (Kosakovsky Pond et al., 2018), are relatively simple to implement and computationally efficient. In such models, clusters are estimated by comparing the genetic diversity of sequence pairs in the dataset, and those most similar are grouped together. However, they lack statistical rigor as they do not provide explicit statistical measures and lack inference of evolutionary history. Specifically, phylogenetic analysis groups sequences that are genetically more similar into clusters and generates a phylogenetic tree where closely related sequences are linked by a common ancestral node. The reliability of clustering can be estimated from how many times clusters of the shuffled sequences share the same common ancestral node. The interpretation of clustering patterns depends on the completeness of samples included (Dasgupta et al., 2019), their representativeness of the population studied, and the methods and algorithms used to reconstruct the phylogeny (Novitsky et al., 2014).

**Fig. 2:**
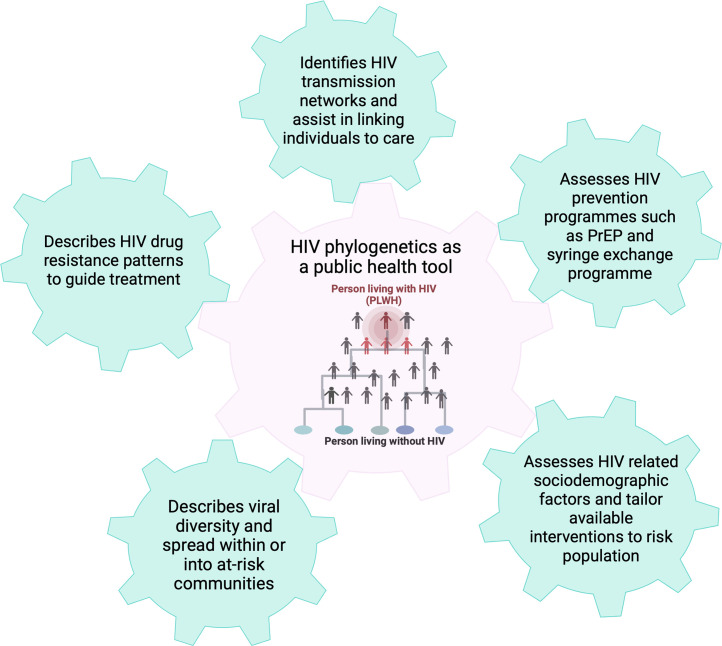
Molecular epidemiology to improve HIV public health interventions. Schematic overview of the different aspects that can be improved by using molecular epidemiology for public health responses. The phylogeny in the center is representative of a molecular cluster that is part of a larger network including who are yet undiagnosed. Molecular epidemiology can aid public health in their prevention efforts and identify risk groups and outbreaks. Molecular epidemiology can also be used to monitor drug resistance and importantly for overall surveillance such as to evaluate the impact of interventions.

The utility of these analyses is increased when sequences are linked to the individuals’ meta data such as sex, age, postcode and risk factors. The combined genome and demographic data allow more targeted public health actions, for example increase access to testing and PrEP specific geographic areas, trigger enhanced community engagement and targeted messaging (also see case studies below for detailed examples). Using this approach, characteristics of HIV transmission, demographic and disease stage of affected individuals within at-risk populations have been described (Hassan et al., 2017; Ragonnet-Cronin et al., 2013; Lundgren et al., 2022; Polotan et al., 2023). The concept has been utilised to understand HIV epidemics among key populations, build informative pictures of networks driving transmission, identify existing transmission networks in communities, and design and assess targeted interventions to stop onward transmission (Fig. 2, supplementary table S1) (German et al., 2016).

For data sampling convenience, several molecular epidemiological studies conducted in the past have opportunistically used HIV-1 sequence data collected for GART for the clinical management of individuals (supplementary table S1). These studies were done in areas of concentrated epidemics and where there is routine GART conducted amongst newly infected patients such as Australia, North America, and Europe as standard of care. In doing so, these studies have identified key features of HIV transmission dynamics.

## 4 Molecular epidemiology for guiding HIV public health interventions

### 4.1 Identifying new and growing clusters

One of the benefits of molecular epidemiology is its ability to identify transmission chains of HIV infection. Patterns of viral transmission within a population can be studied by analysing the genetic sequences of HIV from different individuals. This is particularly useful in identifying and distinguishing between cases that are part of an existing cluster and those that represent a new cluster. Globally, the technique has been used to identify transmission chains among populations (Beyrer et al., 2012). Research from the Swiss HIV Cohort Study showed that phylogenetic analysis of annotated HIV sequence data can be used to predict cluster growth. The authors concluded that knowledge of the nature of past cluster growth was the most important variable in predicting future cluster growth rate and thus, prospective analysis of carefully collected sequence data can serve as a proxy when demographic data is not readily available (Labarile et al., 2023). Such fast-growing clusters were also found in Australia. Two studies from New South Wales identified that 40% of transmission clusters grew continuously over time in the period up from 2004 to 2017 (Di Giallonardo et al., 2019; Di Giallonardo et al., 2021). Identifying transmission clusters in near real-time is pivotal for the effective implementation of molecular epidemiology in the public health response – see case studies Section 5.

### 4.2 Transmission during early stage of infection

HIV diagnoses can lag from month to years after the timepoint of infection as the symptoms of primary infection are often mild and non-specific and many individuals remain asymptomatic (Fiebig et al., 2003). Thus, the initial phase of disease before diagnosis – acute or early stage of infection – is critical in driving transmission dynamics as individuals are unaware of their infection and may have high viral loads (Miller et al., 2010; Hollingsworth et al., 2008; Koopman et al., 1997). Many studies have used molecular epidemiology to estimate the proportion of early stage of infections within transmission clusters to better understand its role in the epidemic spread (Powers et al., 2011; Ragonnet-Cronin et al., 2010; Prins et al., 2023; Verhofstede et al., 2019; Brenner et al., 2007). One study used phylogenetic analysis to identify new clusters of HIV infections in Australia between January 2005 and September 2020 (Chibo et al., 2012). The study concluded that infections among seroconverters (those living with primary – often asymptomatic - HIV infection) contributed to onward transmission. A study from the UK found that 65% of MSM in London were part of a closed transmission network with a quarter of transmissions occurring within six months of primary infection (Lewis et al., 2008). The study highlighted the need to identify transmission networks early to inform effective intervention strategies. A study from Switzerland also found that transmission is more common during the early stage of infection (Marzel et al., 2016). This study underlined the significance of TasP, inclusive of early diagnosis and initiation of continuous treatment. A study from the USA concluded that transmission during the first year of infection was partially linked to high viral load (Little et al., 2014). Similarly, a more recent study from Canada showed that cluster growth was related to transmission from early-stage infection, and this is likely due to HIV strains being more transmissible during this initial period of the infection (Brenner et al., 2021). Thus, early diagnosis and early initiation of ART to reduce viral load is key for reducing transmission rates.

### 4.3 Late diagnosis

Despite improvements in HIV awareness and increased testing rates, HIV diagnoses are still often delayed by months, if not years. Contact tracing is tremendously labor intensive and time-consuming as one needs to assess contacts over longer time periods of potential exposure risk. Hence, larger outbreaks and rapid growing clusters may not be detected for a year or longer, which makes a timely public health response impossible. Thus, a combined effort by the community, the clinicians and the public health units is required to identify unusual spikes in infection rates swiftly, and which could trigger a more targeted prevention path. This could be enhanced by understanding the characteristics associated with rapid cluster growth compared to those associated with cluster termination. The need to increase risk awareness and encourage regular testing amongst risk groups and the promotion of regular testing within communities can help increase early detection of HIV infection and allow small but growing clusters to be revealed through molecular epidemiology.

## 5 Utilizing molecular epidemiological to aid virtual elimination of HIV transmission

### 5.1 Case study from the United States of America

Between 2013 and 2015, 13 rapidly growing HIV transmission clusters amongst predominantly MSMs and other risk populations were found in the three year period (France et al., 2016). Public health authorities confirmed previous detection of only one of those clusters in the affected areas. In 2014, a simulation study using data from San Diego revealed the importance of using molecular analysis to prioritize interventions (Little et al., 2014). In the study, patients were followed up for over one year and comparing their baseline characteristics such as CD4^+^ *T* cell count, viral load, behavioural data, stage of infection and other demographics. Network connections were correlated with the number of onward transmission events. A strong correlation was found between network connection, behavioural data at baseline (e.g. number of unique sex partners and insertive unprotected anal intercourse) and the risk of onward transmission. This underlined the importance of using molecular epidemiology to identify and target risk populations for TasP. Likewise, another study reported a cluster of HIV among PWID in a small town in Indiana (Peters et al., 2016). The study revealed the extreme use of the opiate Oxymorphone among PWID in the community and the contribution of intravenous drug use (IDU) in the introduction and rapid transmission of HIV. This highlighted the need for interventions such as the clean needle and syringe exchange service programs to prevent future outbreaks.

Also, the CDC, has prospectively implemented molecular epidemiology to inform public health interventions (CDC 2020). Identifying and responding to growing HIV transmission clusters has been added to the USA Federal Ending the HIV Epidemic strategy (CDC 2021) by 2030 suggesting HIV cluster detection and response as a key prevention tool (Fauci et al., 2019). The policy is implemented with the CDC developed HIV-TRACE tool that uses genetic distance cut-offs to identify transmission clusters in large datasets (Kosakovsky Pond et al., 2018). Details on the outcomes from this program are summarised by Oster et al. (Oster et al., 2021). Over 145 priority clusters have been identified and reported, informing public health campaigns across USA such as linkage to care and treatment, partner notification, social engagement and PrEP allocation to risk populations (Oster, 2019).

### 5.2 Case study from Canada

The need to track ongoing HIV transmission dynamics and inform public health interventions in real time requires prospective collection and analysis of sequence and meta-data to delineate transmission networks. An implementation case study that utilized phylogenetic analysis of routine clinical data was reported in British Colombia, Canada (Poon et al., 2016). The period required to upload HIV sequences onto the database was significantly reduced to six days allowing for rapid phylogenetic outputs. It was observed that the majority of the clusters comprised of MSMs with drug resistant HIV variants. The individuals were contacted for intensified interventions such as linkage to care, treatment initiation and ART adherence support. Although twelve new cases were reported over a 12-month period, a significant reduction in drug resistant variants was reported suggesting a public health impact from the interventions (Poon et al., 2016). This observation also implicates undiagnosed persons in the onward transmission of HIV to at-risk persons.

## 6 Why does Australia need molecular epidemiology as a pillar in its nationwide HIV-1 prevention strategies?

The Australian HIV Strategy 2018–2022 was a national plan developed by the federal government to guide the country's response to the HIV epidemic. The strategy aimed to reduce the transmission of HIV, improve the health outcomes of people living with HIV, and ultimately work towards the virtual elimination of HIV transmission in Australia by 2022 (AGDOH 2018). The strategy was based on the principles of partnership, innovation, and evidence-based practice, and emphasizes the importance of collaboration between government, health services, community organizations, and people living with HIV. Notably, Australia has reported its lowest number of new HIV diagnoses to date (Kirby, 2023). Perhaps partially driven by the COVID-19 pandemic and strict lockdowns imposed on the population, the number of new HIV notifications among the population declined by a staggering 48%, from 1068 in 2012 to 552 new infections in 2021 (Kirby, 2022). However, this decline was most prominent among Australian-born persons only; 58% decline in HIV-1 infections (4.1 in 2012 to 1.7 in 2021 per 100,000 persons) (Kirby, 2022). Thus, now is the time where a combined effort could lead to the virtual elimination of HIV transmission in Australia. Several efforts have been made towards this goal through institutional partnerships and funding such as the New South Wales HIV Prevention Partnership Project 2022–2026 (Kirby, 2022), individual molecular epidemiological studies (Di Giallonardo et al., 2019; Sacks-Davis et al., 2020; Pinto et al., 2018), the continuation of the needle and syringe program, Healthcare worker training, and the expansion of PrEP prescribers (NSWH, 2020).

The detection of clusters through molecular epidemiology provides a complementary approach to the prevention programs mentioned above. It facilitates the surveillance of ongoing clusters and identification of new outbreaks, thus enabling the identification of gaps in the current prevention by linking the growth of clusters in time to institution of different public health measures. It is worth noting that MSMs living in the outer suburbs of Sydney are found to have an increased relative risk of HIV infection against those in inner Sydney (Grulich et al., 2020). This could be to a lack in safe sex messaging and reduced PrEP access due to lack of knowledge and or stigma and cultural mores, that could also prevent effective contact tracing. Thus, the implementation of effective preventive efforts and the observed decreased HIV prevalence over the years could be compromised by the onward transmission of HIV among these harder-to-reach and harder-to-detect groups within and across states.

## 7 Challenges to molecular epidemiology and possible solutions

Even though molecular epidemiology may be cost-effective and could provide immense public health benefits, in Australia its effective roll-out is limited timely acquisition of data and legal and ethical issues. Using HIV genome data for cluster detection is particularly problematic because phylogenetic methods have been employed in forensics for HIV transmission analysis and subsequent public health investigations and criminal prosecution, especially in areas where the key populations are already under unwanted scrutiny (Sweeney et al., 2017; Abecasis et al., 2018; Jaffe et al., 1994; Birch et al., 2000).

### 7.1 Data collection

One barrier to the implementation of HIV molecular epidemiology in Australia is the lack of jurisdictional uniformity in surveillance systems across States and Territories (Pinto et al., 2018; Callander et al., 2018). The Australian national HIV surveillance system requires notification of all new diagnoses of HIV infection with associated notifiable data that includes patient's age, sex and risk factors for HIV transmission data. While such a differing jurisdictional system exits for epidemiological data (National HIV Registry) (AIHW, 2023), it also does not allow the collection and immediate linkage of genome data. Essentially, patient demographic data and laboratory data for CD4+ *T* cell count and viral load are reported to public health whilst genome data is collected for patient care only. Demographic data is collected quarterly per jurisdictions, and there is no collection of genome data at all outside of the reporting laboratories. Of note, demographic data is reported using a person identifier and only sometimes using full names. Thus, combining genome data and demographic data requires a specialised linkage system that allows to match person identifiers across databases. Without timely access to this data, it is challenging to develop a real-time targeted prevention strategy and monitor its effectiveness.

Developing a national HIV molecular epidemiology strategy that outlines a coordinated approach to data collection and analysis across jurisdictions would overcome these barriers. This strategy should include establishing a national HIV sequence database that collects data from all States and Territories and developing uniform surveillance systems across jurisdictions. Routine collection of HIV sequence data as part of the notifiable data and the development of secure tools to facilitate the reporting, analysis and interpretation of the data collected would simplify processes, as no separate linkage system is required, but would be difficult to implement in the current legal environment. This is because HIV genome data is currently collected for clinical purposes only and is classified as health information (see below ethical and legal issues section*)*. Its use as part of public health would consist of a secondary purpose, which requires individual consent or exemption thereof (Di Giallonardo et al., 2023). Also, investment in training and infrastructure to ensure that public health practitioners have the skills and knowledge to use HIV sequence data effectively is needed.

### 7.2 Ethical and legal issues

Even though clustering methods are unable to infer direction of transmission, concerns about its use in forensic proceedings are well documented (Sweeney et al., 2017; Csete et al., 2022; Field and Sullivan, 1987), and a number of criminal prosecutions have included reference to phylogeny and other clustering methods. The goal of molecular epidemiology is to establish the characteristics of transmission networks and to inform population-based interventions and not identify directionality and person-to-person transmission. The WHO directs the collection and use of public health data only when such data has direct benefit to the population. There is a need to protect the privacy of health data and engage community members when designing programs for implementing molecular epidemiology. In Australia, personal health data is protected by the Privacy Act 1988 and the Australian Privacy Principles (APPs) which require health organisations to protect patients' health data, thus, who can access it and what it can be used for (see above data collection section). There is still a need for broader consultation between government, community members and healthcare providers on the techniques of effective de-identification of patient HIV sequence data to secure standards (Di Giallonardo et al., 2023).

### 7.3 Stigma

Undoubtedly, the stigma experienced by communities affected by HIV and the risk of potential prosecution has caused major information gaps when traditional detection strategies such as testing, partner notification and contact tracing are used (NSWH, 2020). This has been attributed to the reluctance of affected people to provide information on their status and those of their close partners. There is a need to educate communities and government bodies to prohibit misusing molecular epidemiological methods for prosecutions and false attribution of directionality of transmission of infection – the latter cannot be implied using molecular epidemiology. Further, before implementation of clustering analysis in the public health response, ethical issues and concerns need to be addressed in consultation with the affected communities. There is the need to protect health data and engage community members to gain the most out of cluster analyses to help end HIV transmission in those communities.

## 8 Conclusion

The current preventive efforts may be less effective in reducing the HIV prevalence over time due to ongoing transmission among those still undiagnosed or among marginalised population groups that are not accessing these services and therefore have limited access to government-funded interventions such as testing. Employing molecular epidemiology for cluster detection in almost real-time allows identification of clusters when they are small and provides an opportunity for intervention before they grow. This would enable public health authorities to proactively intervene with targeted preventive measures that can help curb further transmissions. However, it is crucial to consider these actions within the framework of ethical and legal principles without the indiscriminate use of patient data other than for public health good.

## Funding

This work was supported by the University International Postgraduate Award (RSRE7061) to BMO and the Medical Research Future Fund (MRFF) Genomics Health Futures Mission - Pathogens Genomics Grant Opportunity (FSPGN000047) to ADK and FDG. The Kirby Institute receives funding from the Australian Government Department of Health, and it is affiliated with the Faculty of Medicine, UNSW Sydney. No pharmaceutical industry grants were received for this study.

## CRediT authorship contribution statement

**Billal M. Obeng:** Formal analysis, Methodology, Visualization, Writing – original draft, Writing – review & editing. **Anthony D. Kelleher:** Conceptualization, Funding acquisition, Supervision. **Francesca Di Giallonardo:** Conceptualization, Supervision, Writing – original draft, Writing – review & editing.

## Declaration of competing interest

The authors declare that they have no known competing financial interests or personal relationships that could have appeared to influence the work reported in this paper.

## Data Availability

No data was used for the research described in the article.

## Appendix Supplementary materials

Image, application 1
