# Supplementary material for: Molecular epidemiology to aid virtual elimination of HIV transmission in Australia
Source: Virus Res. 2024 Jan 11;341:199310. doi: 10.1016/j.virusres.2024.199310 (PMC10825322; doi:10.1016/j.virusres.2024.199310)
Supplement: Supplementary file 1 [file mmc1.docx]

**Table S1. Studies utilizing HIV phylogenetics to delineate transmission dynamics**

| **Region** | **Country** | **Study** | **Data Analysed** | **Phylogeny / clustering methods** | **Predominant Subtype Identified** | **Phylogenetic inference** | **Public health interventions recommended** |
| --- | --- | --- | --- | --- | --- | --- | --- |
| **Australasia** | Australia | [1] | 4,873 *pol* sequences between 2005-2012 | - Maximum likelihood - Genetic Distance of ≤ 1.5% - Bootstrap >98%, ≥3 sequences - 23% of data in clusters | Subtype B, non-B minority | Subtype importation through migration  Clustering linked with subtype B infections, early diagnosis, infections among males and younger people | Establishment of Australian Molecular Epidemiology Network with increased national surveillance methods. |
|  | Australia | [2] | 758 pol sequences 2004-2017 | - Maximum likelihood with global sequence data - Clusters containing ≥80% of local sequences - 66% of data in clusters | CRF01_AE | Most infections acquired outside the country do not lead to local ongoing transmission. Identified an increase in transmission rates among MSM | First study investigating the transmission characterizes for CRF01_AE in NSW. Comprehensive and continuous surveillance is key for monitoring the slow growing clusters and quantify the effectiveness of contact tracing. |
|  | Australia | [3] | 332 *pol* sequences from 2005-2014 | - Maximum likelihood - Genetic Distance of <4.5% - Bootstrap of 95% - 36% of data in clusters | Subtype B | Heterosexual migrants are at higher risk of HIV acquisition  Larger clusters linked to individuals born in Australia and New Zealand | A need for a multifaceted approach to HIV prevention is needed, need to consider changes to eligibility of government funded health coverage for migrants and minor populations. |
|  | Australia | [4] | 2,365 *rt sequences from 2004-2018* | - Maximum likelihood with global sequence data - Clusters being monophyletic for local sequences - 50-77% of data in clusters | All subtypes | Increased clustering among non-B infections over time. | Targeted public health intervention may need modifications to adapt to the changing epidemic. |
|  | Australia | [5] | *pr*, *rt*, *int* sequences from 2919 Subtype B, 473 CRF01_AE from 2004-2018 | - Maximum likelihood with global sequence data - Clusters being monophyletic for local sequences - 53% of B data in clusters - 34% of data in clusters | Subtype B, CRF01_AE as sequence pairs | Clustering linked to different demographics among individuals with B compared CRF01_AE infections. | The need to utilize molecular epidemiological data to inform public health response and prevent onward transmission. |
|  | Thailand | [6] | 135 env gp 160 | - Maximum likelihood - Genetic Distance of ≤3% - Bootstrap of ≥70% - % clustering not specified | CRF01_AE, minority subtypes B, C, CRF01AE/B recombinants | Increased recombinants due to migration, | PrEP among MSM is recommended. |
|  | China | [7] | 1,695 *pol* sequences | - Maximum likelihood and distance method - Genetic distance of ≤4.5% - Bootstrap of 90%, SH-aLRT with 1000 replicates - % clustering not specified | CRF07_BC, CRF55_01B | Post-migration HIV acquisition. | Targeted prevention for foreigners in China is recommended. |

| **Region** | **Country** | **Study** | **Data Analysed** | **Phylogeny / clustering methods** | **Predominant Subtype Identified** | **Phylogenetic inference** | **Public health interventions recommended** |
| --- | --- | --- | --- | --- | --- | --- | --- |
| **North America** | USA | [8] | *pol* sequences from 9 newly-diagnosed patients who had drug-resistant mutations and a previous 53 locally analysed sequences. | - Neighbour-joining - Maximum likelihood - 14% of data in cluster | Not mentioned | - A cluster of newly diagnosed HIV patients with multidrug resistance. - Local surveillance for HIV drug resistance using phylogenetic investigation is important in identifying the outbreak. | The need for primary drug resistance testing in both public and private health departments to detect outbreaks. |
|  | USA | [9] | Total of 673 *pol* sequences (268 San-Diego County cohort, 369 First Choice Program cohort, 36 linked partners) | - Maximum likelihood - Genetic Distance of <1% - 30% of data in clusters | Not mentioned | - Pol sequences is suitable for cluster analysis   Sequences from known recent infections increases size and number of transmission clusters | The need to combining cluster analysis with contact tracing. |
|  | USA | [10] | 3,697 *pol* sequences from 2000-2009 | - Distance method - Genetic Distance of ≤1.5% with ≥2 sequences - % clustering not specified | Not mentioned | Lack of ARV use and high viral load. | Targeted prevention strategy by sex and race. |
|  | USA | [11] | Total of 648 *pol* sequences (478 recently infected and 170 sexual/social contacts)  Between 1996-2011 | - Distance method - Genetic distance of <1.5% - 52.3% of data in clusters | 99% subtype B | High viral load and multiple sex partners are correlated with rapid transmission within the first year of presentation | The need to combining epidemiological analysis with partner services to reduce HIV incidence. |
|  | USA | [12] | Pol sequences from 112 African-born King County’s (KC) residents | - Maximum likelihood - 21% of data in clusters | Subtype C and A1 | African-born residents of KC have lately diagnosed and acquire infection after arrival. | Need to develop culturally appropriate interventions to increase testing among migrants. |
|  | USA | [13] | 24,972 partial pol sequences from 15,246 persons | - Maximum likelihood - Genetic distance of <3.5% and branch support of ≥90% - 0.32% of data in clusters | Subtype C (28.9%), CRF02_AG (24.2%) and subtype A (16.4%) and other non-B subtypes | Increase non-B HIV transmissions is suggestive of the involvement of persons with heterosexual risk behaviors. | Need to survey for transmission clusters to inform interventions based of risk and demographic characteristics. |
|  | USA | [14] | 637 *pol* sequences from 1996-2010 | - Bayesian, Maximum likelihood, Distance method - Genetic Distance of 4%-8% - Bootstrap at ≥90% - 20% of data in clusters | Subtype B with drug resistant mutations, CRF01_AE, Subtypes A and C | Major drivers for clustering were being female, being African American, being Injecting drug user (IDU), and Heterosexual transmission | Need to integrate molecular epidemiologic analyses to identify sources of viral diversity and transmission risks. |
|  | USA | [15] | 156,553 partial *pol* sequences | - HIVTrace distance method - Genetic Distance of 0.5% and 1.5% - 41.8% of data in clusters | Not mentioned | Rapidly growing clusters identified with infections across different states. | National level surveillance needed  Molecular epidemiology can help identify individuals at risk of infection.  PLWHIV not on medical care are candidates for re-engagement.  Need for partner services. |
|  | USA | [16] | *pol* sequences from 22,398 individuals | - Distance method with HIV-TRACE - Genetic distance of 1.5% - 36.3% of data in cluster | Not mentioned | Increased clustering among transgender women. Transgender women are likely to be linked partners of cisgender men who are not not PWID. | The need to expand partner services and link transgender women with HIV to care. |
|  | USA | [17] | *pr* and *rt* sequences from 2775 individuals | - Maximum likelihood - Genetic distance of 0.5%-4.5% and branch support of ≥95% - 17.6% of data in clusters | Subtype B | Evidence of using phylogenetic analysis to identify persons at risk of transmission | The need to direct priority to persons with elevated viraemia especially in the case of stigma and marginalization. The approach provides benefit to the patient and for better health resource allocation. |
|  | Canada | [18] | 32,505 HIV genotype (27850 covered *pol* encoding PR/RT genes, 4655 partial amplicons) | - Maximum likelihood and patristic distance <0.02 - tip with sequences from different infections, at least a sequence from earliest available sample from an individual | Not mentioned | Implicates undiagnosed persons in the onward transmission of HIV-1. | Recommends re-engagement to care services persons who had moved out of care and engaged in high-risk behaviors |
|  | Canada | [19] | 10,945 samples spanning *pr* and *rt* regions | - Distance method - Genetic Distance of <1.5% and 2.5% - Bootstrap >95% Small cluster (2-5 members), large cluster (6-150 members) - ~50% of data in clusters | Subtype B, non-B subtypes | MSM, untreated early-stage infection, younger age group and migration are key to HIV-1 transmission. | Phylodynamic studies needed to identify active, recurrent large cluster networks. Need for lower barriers to testing and PrEP uptake. |
|  | Canada | [20] | 105 *pol* sequences | - Neighbour-joining method - Genetic distance of <1% and bootstrap support of >97% - 12.3% of data in clusters | Subtypes B, AG, G | Identification of clusters of individuals from same country of birth | The need for culturally appropriate HIV prevention and treatment services in Quebec, Canada |

| **Region** | **Country** | **Study** | **Data Analysed** | **Phylogeny / clustering methods** | **Predominant Subtype Identified** | **Phylogenetic inference** | **Public health interventions recommended** |
| --- | --- | --- | --- | --- | --- | --- | --- |
| **Africa** | South Africa | [21] | 375 samples from *gag* p24 and partial *pol* fragments between 1998-2004 | - Maximum likelihood - Bootstrap support >90% - % clustering not specified | Subtypes A1, B, G | Viral recombinant spread. The contribution of socio-political situations and migration in Southern Africa to the spread of HIV-1. | Molecular epidemiology is important to track factors that drive transmission and assess existing interventions. |
|  | South Africa | [22] | 200 *pol* sequences, 2294 Subtype C reference sequences | - Maximum likelihood - Genetic Distance of 1%-4.5%, Bootstrap of 90% - 26% of data in clusters | Subtype C | High genetic diversity and no structural phylogeny.  No statistical links between clustering and demographics. | Molecular epidemiology is important to track HIV-1 subtypes and their transmission |
|  | Nigeria | [23] | 672 *pol* sequences | - Maximum likelihood and distance method - Genetic Distance of 1.5% - % clustering not specified | CRF02_AG, subtype G | Men who have Sex with men (MSM) represents a previously hidden reservoir of new HIV-1 strains including CRF95_02B and CRF56_cpx. | Different HIV subtypes to be considered in global efforts for vaccine selection and development. Need for continued epidemiological surveillance. |
|  | Uganda | [24] | 2,531 partial *pol* sequences | - Phyloscanner - % clustering not specified | Any | 82% of clusters were pairs. Reconstruction of recipient-source pairs showed more transmission from general population to fishing communities than vice versa. | Targeted intervention for risk group should be expanded to surrounding areas. |
|  | Uganda | [25] | 2,017 partial *pol* sequences | - Maximum likelihood - ClusterPicker 4.5% - Bootstrap of >95% - 17.2% of data in clusters | Subtype A1, D, C, Recombinants | Identified different network parameters for different population groups. Most transmission linked to a core group. | Targeted intervention for core groups most at risk must be timely. |
|  | Botswana | [26] | 3,832 partial *pol* sequences | - ClusterPicker 0.05%-3.5% - HIV-TRACE 0.05%-3.5% - 14% of data in clusters | Subtype C | Transmission occurs among similarly aged partners within and between communities but is more common into intervention communities than control communities. | Widely distributed and accessible interventions are needed to reduce transmission. |

| **Region** | **Country** | **Study** | **Data Analysed** | **Phylogeny / clustering methods** | **Predominant Subtype Identified** | **Phylogenetic inference** | **Public health interventions recommended** |
| --- | --- | --- | --- | --- | --- | --- | --- |
| **Europe** | Italy | [27] | 3,499 *pol* sequences | - Maximum likelihood and HIV-TRACE - Genetic distance of <0.1 substitutions/site - Node support >90% - 20.7% of data in cluster | mainly subtype B | Clustering linked to infections among MSM, among individuals of Italian origin, and being of younger age. Infections among migrants rarely lead to clustering. | Phylogenetics is useful for monitoring the HIV epidemic. |
|  | France | [28] | 547 *rt* sequences from 2008 to 2011 | - Distance method, Maximum likelihood - Genetic Distance of <1.5%, Bootstrap >98% - 19.7% of data in clusters | Subtype B | Clustering linked to infections from very early diagnoses and from younger individuals. Low proportion of clustering may be due to infections from undiagnosed. | Treatment as Prevention (TasP) recommended. |
|  | Switzerland | [29] | 19,604 *pol* sequences | - Maximum likelihood - Genetic Distances of 1%-2.5%, Bootstrap of 50-100% - % clustering not specified | Subtype B with minority non-B subtype | Transmission more common in the first year of infection and after ART interruption | TasP needs to be accompanied by other interventions (early diagnosis and adherence) |
|  | United Kingdom | [30] | 14,000 pol sequences from 2007-2009 | - Maximum likelihood - Genetic Distance of ≤4.5%, 90% bootstrap support - % clustering not specified | Subtypes A1, C, D, G | Higher growth rate amongst MSM, PIWD with non-B subtype infections | Expansion of prevention efforts to include all risk groups and subtypes. |
|  | United Kingdom | [31] | 2572 *pol* sequences | - Pairwise distance - Genetic distance 1-2.5% - 19.5% of data in clusters | Subtype C | Large cluster of 104 sequences among PWID. | PWID are still at risk for infection despite public health interventions. Increased availability to testing and injecting equipment is needed. |
|  | Poland | [32] | 966 *pol* sequences | - Maximum likelihood - Genetic Distance of <3%, Bootstrap >90% - 33.2% of data in clusters | Subtype B | Increased clustering and inter-regional networks amongst Polish MSMs | Active testing and treatment amongst MSMs are needed. |

**References**

1. Castley A, Sawleshwarkar S, Varma R, Herring B, Thapa K, Dwyer D, et al. A national study of the molecular epidemiology of HIV-1 in Australia 2005–2012*.* PLoS One. 2017; 12(5):e0170601.

2. Di Giallonardo F, Pinto AN, Keen P, Shaik A, Carrera A, Salem H, et al. Limited sustained local transmission of HIV-1 CRF01_AE in New South Wales, Australia*.* Viruses. 2019; 11(5):482.

3. Sacks-Davis R, Chibo D, Peach E, Aleksic E, Crowe SM, El Hayek C, et al. Phylogenetic clustering networks among heterosexual migrants with new HIV diagnoses post-migration in Australia*.* PLoS One. 2020; 15(9):e0237469.

4. Di Giallonardo F, Pinto AN, Keen P, Shaik A, Carrera A, Salem H, et al. Increased HIV Subtype Diversity Reflecting Demographic Changes in the HIV Epidemic in New South Wales, Australia*.* Viruses. 2020; 12(12):1402.

5. Di Giallonardo F, Pinto AN, Keen P, Shaik A, Carrera A, Salem H, et al. Subtype‐specific differences in transmission cluster dynamics of HIV‐1 B and CRF01_AE in New South Wales, Australia*.* J Int AIDS Soc. 2021; 24(1):e25655.

6. Chang D, Sanders‐Buell E, Bose M, O'Sullivan AM, Pham P, Kroon E, et al. Molecular epidemiology of a primarily MSM acute HIV‐1 cohort in Bangkok, Thailand and connections within networks of transmission in Asia*.* J Int AIDS Soc. 2018; 21(11):e25204.

7. Yan H, Wu H, Xia Y, Huang L, Liang Y, Li Q, et al. Acquisition and transmission of HIV-1 among migrants and Chinese in Guangzhou, China from 2008 to 2012: Phylogenetic analysis of surveillance data*.* Infect Genet Evol. 2021; 92:104870.

8. Buskin SE, Ellis GM, Pepper GG, Frenkel LM, Pergam SA, Gottlieb GS, et al. Transmission Cluster of Multiclass Highly Drug-Resistant HIV-1 Among 9 Men Who Have Sex With Men in Seattle/King County, WA, 2005− 2007*.* J Acquir Immune Defic Syndr. 2008; 49(2):205.

9. Smith DM, May S, Tweeten S, Drumright L, Pacold ME, Pond SLK, et al. A public health model for the molecular surveillance of HIV transmission in San Diego, California*.* AIDS. 2009; 23(2):225.

10. Aldous JL, Pond SK, Poon A, Jain S, Qin H, Kahn JS, et al. Characterizing HIV transmission networks across the United States*.* Clin Infect Dis. 2012; 55(8):1135-43.

11. Little SJ, Kosakovsky Pond SL, Anderson CM, Young JA, Wertheim JO, Mehta SR, et al. Using HIV networks to inform real time prevention interventions*.* PLoS One. 2014; 9(6):e98443.

12. Kerani RP, Herbeck JT, Buskin SE, Dombrowksi JC, Bennett A, Barash E, et al. Evidence of local HIV transmission in the African community of King County, Washington*.* J Immigr Minor Health. 2017; 19:891-6.

13. Dennis AM, Hue S, Learner E, Sebastian J, Miller WC,Eron JJ. Rising prevalence of non-B HIV-1 subtypes in North Carolina and evidence for local onward transmission*.* Virus Evol. 2017; 3(1):vex013.

14. Dalai SC, Junqueira DM, Wilkinson E, Mehra R, Kosakovsky Pond SL, Levy V, et al. Combining phylogenetic and network approaches to identify HIV-1 transmission links in San Mateo County, California*.* Front Microbiol. 2018:2799.

15. Oster AM, France AM, Panneer N, Ocfemia MCB, Campbell E, Dasgupta S, et al. Identifying clusters of recent and rapid HIV transmission through analysis of molecular surveillance data*.* J Acquir Immune Defic Syndr. 2018; 79(5):543.

16. Ragonnet-Cronin M, Hu YW, Morris SR, Sheng Z, Poortinga K,Wertheim JO. HIV transmission networks among transgender women in Los Angeles County, CA, USA: a phylogenetic analysis of surveillance data*.* Lancet HIV. 2019; 6(3):e164-e72.

17. Kassaye SG, Grossman Z, Vengurlekar P, Chai W, Wallace M, Rhee S-Y, et al. Insights into HIV-1 Transmission Dynamics Using Routinely Collected Data in the Mid-Atlantic United States*.* Viruses. 2022; 15(1):68.

18. Poon AF, Gustafson R, Daly P, Zerr L, Demlow SE, Wong J, et al. Near real-time monitoring of HIV transmission hotspots from routine HIV genotyping: an implementation case study*.* Lancet HIV. 2016; 3(5):e231-8.

19. Brenner BG, Ibanescu R-I, Osman N, Cuadra-Foy E, Oliveira M, Chaillon A, et al. The Role of Phylogenetics in Unravelling Patterns of HIV Transmission towards Epidemic Control: The Quebec Experience (2002–2020)*.* Viruses. 2021; 13(8):1643.

20. Park H, Brenner B, Ibanescu R-I, Cox J, Weiss K, Klein MB, et al. Phylogenetic Clustering among Asylum Seekers with New HIV-1 Diagnoses in Montreal, QC, Canada*.* Viruses. 2021; 13(4):601.

21. Wilkinson E, Engelbrecht S,De Oliveira T. History and origin of the HIV-1 subtype C epidemic in South Africa and the greater southern African region*.* Sci Rep. 2015; 5(1):1-12.

22. Sivay MV, Hudelson SE, Wang J, Agyei Y, Hamilton EL, Selin A, et al. HIV-1 diversity among young women in rural South Africa: HPTN 068*.* PLoS One. 2018; 13(7):e0198999.

23. Billings E, Kijak GH, Sanders-Buell E, Ndembi N, O’Sullivan AM, Adebajo S, et al. New subtype B containing HIV-1 circulating recombinant of sub-Saharan Africa origin in Nigerian men who have sex with men*.* J Acquir Immune Defic Syndr. 2019; 81(5):578.

24. Bbosa N, Ssemwanga D, Ssekagiri A, Xi X, Mayanja Y, Bahemuka U, et al. Phylogenetic and demographic characterization of directed HIV-1 transmission using deep sequences from high-risk and general population cohorts/groups in Uganda*.* Viruses. 2020; 12(3):331.

25. Bbosa N, Ssemwanga D, Nsubuga RN, Kiwanuka N, Bagaya BS, Kitayimbwa JM, et al. Phylogenetic Networks and Parameters Inferred from HIV Nucleotide Sequences of High-Risk and General Population Groups in Uganda: Implications for Epidemic Control*.* Viruses. 2021; 13(6):970.

26. Magosi LE, Zhang Y, Golubchik T, DeGruttola V, Tchetgen ET, Novitsky V, et al. Deep-sequence phylogenetics to quantify patterns of HIV transmission in the context of a universal testing and treatment trial–BCPP/Ya Tsie trial*.* Elife. 2022; 11:e72657.

27. Fabeni L, Alteri C, Orchi N, Gori C, Bertoli A, Forbici F, et al. Recent transmission clustering of HIV-1 C and CRF17_BF strains characterized by NNRTI-related mutations among newly diagnosed men in central Italy*.* PLoS One. 2015; 10(8):e0135325.

28. Robineau O, Frange P, Barin F, Cazein F, Girard P-M, Chaix M-L, et al. Combining the estimated date of HIV infection with a phylogenetic cluster study to better understand HIV spread: application in a Paris neighbourhood*.* PLoS One. 2015; 10(8):e0135367.

29. Marzel A, Shilaih M, Yang W-L, Böni J, Yerly S, Klimkait T, et al. HIV-1 transmission during recent infection and during treatment interruptions as major drivers of new infections in the Swiss HIV Cohort Study*.* Clin Infect Dis. 2016; 62(1):115-22.

30. Ragonnet-Cronin M, Lycett SJ, Hodcroft EB, Hué S, Fearnhill E, Brown AE, et al. Transmission of non-B HIV subtypes in the United Kingdom is increasingly driven by large non-heterosexual transmission clusters*.* J Infect Dis. 2016; 213(9):1410-8.

31. Ragonnet-Cronin M, Hué S, Hodcroft EB, Tostevin A, Dunn D, Fawcett T, et al. Non-disclosed men who have sex with men in UK HIV transmission networks: phylogenetic analysis of surveillance data*.* Lancet HIV. 2018; 5(6):e309-e16.

32. Parczewski M, Leszczyszyn-Pynka M, Witak-Jędra M, Szetela B, Gąsiorowski J, Knysz B, et al. Expanding HIV-1 subtype B transmission networks among men who have sex with men in Poland*.* PLoS One. 2017; 12(2):e0172473.
